# Editorial: Soil microorganisms under ecological planting

**DOI:** 10.3389/fmicb.2023.1227230

**Published:** 2023-08-08

**Authors:** Pengfa Li, Jia Liu, Muhammad Saleem, Ming Liu

**Affiliations:** ^1^State Key Laboratory of Soil and Sustainable Agriculture/Institute of Soil Science, Chinese Academy of Sciences, Nanjing, China; ^2^Department of Microbiology, College of Life Sciences, Nanjing Agricultural University/Key Laboratory of Agricultural and Environmental Microbiology, Ministry of Agriculture and Rural Affairs, Nanjing, China; ^3^National Engineering and Technology Research Center for Red Soil Improvement/Institute of Soil & Fertilizer and Resources & Environment, Jiangxi Academy of Agricultural Sciences, Nanchang, China; ^4^Department of Biological Sciences, Alabama State University, Montgomery, AL, United States; ^5^University of Chinese Academy of Sciences, Nanjing/University of Chinese Academy of Sciences, Beijing, China

**Keywords:** soil sustainability, plant productivity, crop rotation, intercropping, soil microorganism, element cycle, plant-microbe interactions

As the global human population is expected to reach nine billion by 2050, ensuring stable and sufficient food, poultry, and fiber supplies is a major challenge. To increase food production, chemical fertilizers and pesticides have been widely used in agriculture, but they have caused serious harm to the environment and human health. Therefore, it is urgent to adopt sustainable agricultural practices that can improve and maintain crop yield and quality. One such practice is ecological planting, which combines traditional methods of crop rotation, intercropping, or relay intercropping with modern agricultural technology, based on the growth rhythm of different crops on a unit area of land. This way, natural and biological resources, such as light, heat, water, and fertilizer, can be used more efficiently and fully to achieve higher yield and economic and ecological benefits.

Soil microorganisms link above-ground and below-ground ecosystems and play an important role in soil nutrient cycling, soil-borne disease control, and other ecosystem processes. Ecological planting can increase above-ground plant diversity and subsequently influence below-ground microorganisms. However, the effects of above-ground crops on below-ground microorganisms are still unclear and contradictory. For example, how microorganisms drive the cycling of C, N, P, S, and other elements or pollutants under different cropping patterns is a cutting-edge topic that is far from clear. Moreover, the complex plant–microbe interactions, such as the relationship between plant disease, soil-borne phytopathogens, and plant-beneficial microbes, under ecological planting remain poorly understood.

In this Research Topic, 13 articles were published, providing a deeper understanding of microbial response, process, and function under ecological planting. We aim to elucidate the response of the under-ground microbial process and ecological function, the interaction mechanisms between beneficial microbes and pathogens, and the changes in microbial community and metabolites under different cropping patterns with different management strategies. These findings will advance our understanding of soil microbial ecological processes and functions under ecological planting.

Xiang et al. studied the effects of green manure (hairy vetch) and peanut straw on soil fertility, peanut yield, and the AMF community in Ultisol dryland. The study found that the application of green manure improved soil nutrients and peanut yield and promoted the positive feedback relationship between peanut and the AMF community by increasing soil AMF abundance and network stability. In rice cultivation, leguminous crop rotation can increase soil productivity, but little is known about the role of microorganisms in soil productivity under leguminous crop rotation. Xia et al. found that Chinese milk vetch rotation can enrich key microbial groups with potential phosphorus dissolution ability, increase soil available phosphorus content, and ultimately increase crop yield. The appropriate amount of Chinese milk vetch instead of chemical fertilizer can also improve the nitrogen cycle function of key ecological bacterial communities in soil (Lv et al.). Reasonable nitrogen application and organic agriculture, including Chinese milk vetch returning, can also effectively improve soil microbial diversity and soil fertility in wheat–rice rotation (Xu et al.).

Besides the farmland ecosystem, Sun et al. studied soil microbial changes under the *Cinnamomum camphora* canopy and between *C. camphora* trees. The study showed that *C. camphora* planting can improve soil fertility in subtropical regions and promote the transformation of soil microbial communities from *oligotrophic* (K-strategy) to *eutrophic* bacteria (r-strategy). The effects of canopy nitrogen addition and understory management on soil microbial communities in Chinese fir forests were also investigated. The results showed that short-term canopy nitrogen addition changed soil bacterial and fungal communities, leading to a stronger response in the surface and middle soil layers than in the deep soil layer, while understory removal may enhance the effect on soil bacterial abundance (Xi et al.).

Regarding the inhibition of soil-borne diseases and the elimination of soil pollution, studies have found that when tomato plants are infected by pathogens, some harmful biological pathways such as quorum sensing are significantly enriched, while some beneficial biological pathways such as streptomycin biosynthesis are depleted. These findings extend our understanding of plant–microbe interactions and provide new clues for understanding the underlying mechanisms of interactions between plant microbial communities and pathogens (Kuang et al.). Li Q.-M. et al. found that rotation significantly changed the composition of bacterial and fungal communities, increased potentially plant-beneficial fungi, and reduced the relative abundance of potentially phytopathogenic fungi. You et al. found that *Glomus mosseae* affected the rhizosphere microbial community by regulating soil properties and greatly enhanced the cadmium remediation ability of *Solanum nigrum*.

With respect to biological network interactions and microbial and metabolite interactions, a 10-year field experiment revealed that the potential predation pressure of nematodes on bacteria may enhance bacterial abundance, which in turn has a positive effect on carbon fixation. The interaction between nematodes and microorganisms plays an important role in regulating carbon dynamics under the return of organic matter (Shi et al.). Li M. et al. found that different compounds in root exudates were the main driving factors affecting the microbial community in the *Populus* rhizosphere. The key taxa of the rhizosphere microbial community are very important for maintaining the rhizosphere stability. Root exudates had a stronger effect on key bacteria than on dominant bacteria and fungi. Zhang et al. found that the use of enzymatic fermented soybean fertilizer can improve soil nutrition, regulate related microbial communities, and have a positive impact on the lipid metabolites of new tea shoots, which provides feasible technical guidance for the use of soybean as an advanced fertilizer to produce high-quality tea.

Moreover, the effects of habitat fragmentation on soil microbial biomass, diversity, and community assembly under two typical plants in the mainland–island system were also investigated (Wu et al.).

For future research on microbial processes under ecological planting, the following aspects should also be considered. First, unraveling the complex plant–microorganism interaction mechanisms under the ecological planting pattern. Second, developing and optimizing ecological planting strategies for the synergistic improvement of soil quality and plant productivity. Third, understanding the transformation of soil carbon and carbon sequestration and emission reduction under ecological planting in the context of climate change. Fourth, achieving the One Health concept for soil, plants, microorganisms, the environment, and human health by using ecological planting.

## Author contributions

PFLi and MLiu revised the manuscript and wrote the final version of the manuscript. All authors listed have made a substantial, direct, and intellectual contribution to the work and approved it for publication.

